# COVID-19 und Gastroenterologie

**DOI:** 10.1007/s11377-023-00684-5

**Published:** 2023-03-15

**Authors:** Martina Müller-Schilling, Andreas Stallmach

**Affiliations:** 1 Klinik und Poliklinik für Innere Medizin I, Gastroenterologie, Hepatologie, Endokrinologie, Infektiologie und Rheumatologie, Universitätsklinikum Regensburg, 93042 Regensburg, Deutschland; 2 Klinik für Innere Medizin IV, Gastroenterologie, Hepatologie, Infektiologie, Universitätsklinikum Jena, 07747 Jena, Deutschland

Die COVID-19-Pandemie hat die Welt verändert. Diese Ausgabe der Zeitschrift *Die Gastroenterologie* legt ihren Fokus auf die Auswirkungen von COVID-19 auf die Gastroenterologie und die Hepatologie.

*K. Schütte und C. Schulz* geben einen Überblick über die gastroenterologischen Aspekte der COVID-19-Pandemie. Ein signifikanter Teil von PatientInnen mit SARS-CoV-2-Infektion berichtet über gastrointestinale Symptome. So zeigt eine jüngere prospektive multizentrische Studie, in die wegen COVID-19 hospitalisierte PatientInnen eingeschlossen wurden, eine Anorexie bei 49,8 %, Diarrhö bei 39,4 %, Übelkeit und Erbrechen bei 27,4 % und abdominelle Schmerzen bei 20,7 % der PatientInnen zum Zeitpunkt der stationären Aufnahme [1]. Die AutorInnen besprechen darüber hinaus die sektorenübergreifend signifikanten Auswirkungen der COVID-19-Pandemie auf die Versorgungsstrukturen in der Gastroenterologie. Neben den Herausforderungen für die Aufrechterhaltung einer guten medizinischen Versorgung aller PatientInnen sehen die AutorInnen zukünftige Herausforderungen insbesondere darin, die Tätigkeit im Gesundheitssystem durch strukturelle, inhaltliche und psychologische Unterstützung der AkteurInnen attraktiv zu halten und auch unter den Umständen einer Pandemie eine qualitativ hochwertige Ausbildung im pflegerischen und ärztlichen Bereich sicherzustellen.

Mit diesen Herausforderungen und Folgen spezifisch für die Endoskopie und das Endoskopiepersonal beschäftigt sich der Beitrag von *P. Mester, A. Kandulski et al.* Endoskopische Untersuchungen des oberen Gastrointestinaltrakts können als aerosolgenerierende Prozeduren eingeordnet werden. Die Verwendung von persönlicher Schutzausrüstung („personal protective equipment“, PPE) als infektionspräventive Maßnahme in der Endoskopie ist effektiv und wahrscheinlich in ihrer Wirksamkeit höher einzuschätzen als präendoskopische Teststrategien. Die AutorInnen zeigen, dass die Präventionsstrategien während der Pandemie erfolgreich und die Infektionsraten des Personals in der Endoskopie durch die eingeleiteten nun evidenzbasierten Maßnahmen sehr gering waren.

COVID-19-Pandemie beeinflusst das Leben von PatientInnen mit CED in großem Umfang

Die COVID-19-Pandemie beeinflusst das Leben von PatientInnen mit chronisch- entzündlichen Darmerkrankungen (CED) in großem Umfang. Betroffene und ihre Angehörige haben viele Fragen zum Erkrankungsrisiko, zum Verlauf einer potenziellen SARS-CoV-2-Infektion, zur Impfung oder auch zum Einfluss der CED-spezifischen Therapie. *N. Teich und A. Stallmach* beantworten diese Fragen und fassen die neuesten Erkenntnisse zu CED und COVID-19 und die Diskussion zum Impfansprechen zusammen. Sie zeigen, dass PatientInnen mit chronisch-entzündlichen Darmerkrankungen kein erhöhtes Risiko einer SARS-CoV-2-Infektion oder einer schweren COVID-19-Erkrankung tragen. Hohes Alter, männliches Geschlecht, signifikante Komorbiditäten und eine systemische Steroidtherapie erhöhen jedoch dieses Risiko. CED-PatientInnen weisen bei begleitender COVID-Erkrankung häufiger als Nicht-CED-PatientInnen gastrointestinale Symptome auf und es sollte daher bei einem CED-Schub auch an eine SARS-CoV-2-Infektion gedacht werden. PatientInnen mit einer stabilen Remission haben ein deutlich niedrigeres Risiko eines schweren COVID-19-Verlaufs als PatientInnen im akuten CED-Schub oder im chronisch-aktiven Verlauf. Daher soll – auch im Fall einer SARS-CoV-2-Infektion – eine remissionserhaltende Therapie beibehalten werden. Alle PatientInnen mit CED sollten gegen SARS-CoV‑2 geimpft werden, auch wenn das Impfansprechen medikationsabhängig eingeschränkt sein kann.

*S. Schmid et al.* besprechen die Auswirkung von COVID-19 auf die Leber. Eine hepatische Beteiligung ist bei COVID-19 häufig und von hoher klinischer Relevanz. Erhöhte Leberwerte sind wichtige Prädiktoren der Prognose von PatientInnen mit SARS-CoV-2-Infektion. Differenzialdiagnosen für Leberwerterhöhungen bei COVID-19, wie andere Virusinfektionen, medikamentös induzierte Leberschädigung sowie autoimmune, metabolische und weitere Lebererkrankungen, sollten stets abgeklärt werden. Bei kritisch kranken PatientInnen ist an die Entwicklung einer sekundär sklerosierenden Cholangitis (COVID-SSC) zu denken. Hier ist die frühzeitige Durchführung einer ERCP mit Extraktion von „casts“ eine wichtige Therapieoption. In schweren Fällen sollte eine Lebertransplantation erwogen werden. Bei PatientInnen mit vorbestehender Lebererkrankung oder einer Leberzirrhose kann es durch COVID-19 zu einer Dekompensation mit Aszites und hepatischer Enzephalopathie bis hin zum akut-auf-chronischen Leberversagen (ACLF) kommen.

*L. Wiering und F. Tacke* geben einen praxisnahen Überblick über die aktualisierte S1-Leitlinie zur Versorgung von Lebertransplantierten während der COVID-19-Pandemie. Basis dieser Übersicht ist die aktualisierte Leitlinie (Stand 15.06.2022) der DGVS und DGAV. Die AutorInnen stellen dar, dass Lebertransplantationsprogramme inkl. Evaluation, Organspenden und Nachsorge möglichst unverändert während einer Pandemie fortgeführt werden sollen, da sie eine lebensrettende Therapieoption darstellen. Die mittlerweile zur Verfügung stehende Immunisierung sowie die antivirale/immunmodulierende Therapie erlauben eine deutlich verbesserte Prävention und Therapie von COVID-19 bei Lebertransplantierten. Eine frühzeitige Erkennung durch engmaschige Testung ist von hoher Wichtigkeit. Weiterhin erlauben die verbesserten pharmakologischen Optionen unter Risiko-Nutzen-Abwägung auch eine Transplantation von positiven Spendern oder Empfängern.

Mit diesen verbesserten pharmakologischen Optionen und der Therapie von COVID-19 unter Berücksichtigung von Lebererkrankungen beschäftigt sich der Beitrag von *M. Cornberg und C. Dietz-Fricke*. Patienten mit chronischen Lebererkrankungen, insbesondere mit Leberzirrhose, sowie immunsupprimierte Personen nach Lebertransplantation scheinen generell ein erhöhtes Infektionsrisiko zu haben, was sich in einer erhöhten Mortalität niederschlägt. Dies gilt auch für die SARS-CoV-2-Infektion, bei der insbesondere PatientInnen mit Leberzirrhose ein hohes Risiko für einen schweren COVID-19-Verlauf haben. Die COVID-19-Pandemie hat zu einer außergewöhnlich raschen Entwicklung von Impfstoffen, prophylaktischen und therapeutischen Antikörpern sowie zur Erprobung neuer und bereits für andere Indikationen zugelassener Arzneimittel geführt. Die Autoren fassen diese Therapiekonzepte unter besonderer Berücksichtigung von PatientInnen mit chronischen Lebererkrankungen und Patienten nach Lebertransplantation zusammen.

Während der überwiegende Anteil der PatientInnen nach einer SARS-CoV-2-Infektion ohne Folgen gesundet, kommt es bei einem Teil der PatientInnen zu Folgeerscheinungen, die Monate andauern können. Nach Schätzungen der Weltgesundheitsorganisation (WHO) klagen 10–20 % der SARS-CoV-2-Infizierten über bleibende oder neu aufgetretene Beschwerden nach der Akutphase der Infektion, die als Post-COVID-Syndrom bezeichnet werden [2–5].

Hier verdeutlicht der Beitrag von *S. Quickert et al.*, dass – auch wenn nun ein Übergang des pandemischen Verlaufs von COVID-19 in einen endemischen Verlauf zu erwarten ist – wir uns medizinisch noch lange mit dem Virus und den daraus resultierenden Folgen beschäftigen müssen. Obwohl die Schwere der Krankheitsverläufe mit den neu aufgetretenen Virusvarianten und der zunehmenden Immunisierung der Bevölkerung abgenommen hat, stieg im Gegensatz dazu der Anteil der gastroenterologischen Symptome, insbesondere Übelkeit, Erbrechen und Diarrhö, im Verlauf der Pandemie an. Daher sollte eine Infektion mit SARS-CoV‑2 immer mit in differenzialdiagnostische Überlegungen einbezogen werden, wenn sich PatientInnen mit unklaren Durchfällen vorstellen. Aufgrund der vielfältigen Symptome, die bei einem Post-COVID-Syndrom auftreten können, sollte ferner die Erhebung einer vorangegangen SARS-CoV-2-Infektion Teil der Anamnese bei PatientInnen sein, die sich mit neuaufgetretenen unklaren gastroenterologisch-hepatologischen Erkrankungen vorstellen.

Wir freuen uns über die hohe Qualität der Beiträge und danken allen AutorInnen der aktuellen Ausgabe der Zeitschrift *Die Gastroenterologie*, dass Sie unserer Einladung nachgekommen sind. Unseren LeserInnen wünschen wir Erkenntnisgewinn für die tägliche Arbeit in Klinik und Praxis.
